# Cross-sectional epidemiological assessment of lymphatic filariasis situation in areas under post-mass drug administration surveillance and the associated risk of transmission in the context of migrants in India: a study protocol

**DOI:** 10.1136/bmjopen-2025-116111

**Published:** 2026-01-30

**Authors:** Adinarayanan Srividya, Raja J Dinesh, Muhammed Jabir M M, Manikandan Kishanthini, Vishal Dogra, Bhupendra Tripathi, Rinku Sharma, Tanu Jain, Manju Rahi

**Affiliations:** 1 ICMR - Vector Control Research Centre, Puducherry, India; 2 Gates Foundation, New Delhi, India; 3 Infectious Diseases & Vaccine Delivery, Gates Foundation, New Delhi, India; 4 National Center for Vector Borne Diseases Control, Government of India Ministry of Health & Family Welfare, New Delhi, India

**Keywords:** EPIDEMIOLOGIC STUDIES, INFECTIOUS DISEASES, Tropical medicine, PUBLIC HEALTH

## Abstract

**Abstract:**

**Introduction:**

India targets to eliminate lymphatic filariasis (LF) in alignment with the global goals. By 2024, 106 out of a total of 345 endemic districts have passed all three serial transmission assessment surveys (TAS) and are under post-mass drug administration (MDA) surveillance for a variable period. However, the current epidemiological situation of LF is not known in these districts. With increased mobility of population from the endemic districts currently under MDA to these post-MDA areas, resurgence of LF in these areas cannot be ruled out. Therefore, a study is planned to understand the current LF status in areas under post-MDA surveillance with the following objectives: (1) To assess the epidemiological situation of LF in terms of human and vector infection prevalence in selected evaluation units (EUs) under different durations of post-MDA phase and (2) to estimate the filarial infection (in terms of filarial antigen and microfilaria) among migrants (from endemic districts) in these EUs.

**Methods and analysis:**

This cross-sectional study will measure the filarial infection in (1) adult population aged ≥20 years (following the WHO 2025 protocol for monitoring and evaluation of MDA) among general population (n=3150 per EU), (2) migrant population (aged 2 years and above) in the post-MDA area originating from endemic areas (n=1000 per EU) and (3) vectors (n=7500 per EU) using molecular xenomonitoring (MX) to confirm sustenance of transmission interruption or identify any potential risk of resurgence in three EUs under post-MDA phase. In one MDA-naive EU that shares borders with endemic districts, filarial infection status will be assessed in (1) school children aged 9–14 years (as per WHO mini-TAS protocol, n=480), (2) migrants (aged 2 years and above) from endemic areas (n=1000) and (3) vectors (n=7500). EU-wide prevalence of microfilaria, circulating filarial antigen and vector infection rates with 95% CIs will be estimated. Multivariate logistic regression analysis will be carried out to find factors associated with LF positivity. In addition, knowledge, attitude and practice surveys will also be conducted among the adult migrants (n=1000 per EU). Thirty in-depth interviews will be conducted among the migrants, local community and health workers (in each EU) and the results will be suitably analysed and triangulated. The study results will enable the national programme to confirm sustenance of transmission interruption or assist in taking a decision to reinitiate MDA in these areas under post-MDA surveillance. It will also enable devising specific strategies to treat migrants.

**Ethics and dissemination:**

This study has been approved by the institutional ethics committee (IHEC 03-0824/N/F). A workshop will be held with all stakeholders to disseminate the study findings.

**STRENGTHS AND LIMITATIONS OF THIS STUDY**

- A mixed-method design integrating epidemiological, behavioural and entomological aspects to assess risk of resurgence in the post-mass drug administration (MDA) areas.
- Use of molecular xenomonitoring (MX), a sensitive tool capable of detecting low-level transmission in vector mosquitoes.
- Risk of recall bias and low risk perception for lymphatic filariasis in the community as MDA was stopped several years’ back.
- Engaging migrant populations may pose operational challenges.

## Introduction

 Lymphatic filariasis (LF) is a neglected tropical disease targeted for global elimination as a public health problem (EPHP) by 2030.^1^ Globally, 485 million people are at risk of infection and require preventive chemotherapy.^2^ The Global Programme to Eliminate Lymphatic Filariasis, launched in 2000 was built on two strategic pillars: (1) interruption of transmission through mass drug administration (MDA) of antifilarial drugs (either two drug regimen with diethylcarbamazine/ivermectin and albendazole-DA/IA or three drug regimen with ivermectin, diethylcarbamazine and albendazole-IDA or single drug with albendazole in areas coendemic for loiasis) and (2) alleviation of suffering among those diseased by providing an essential package of care to manage and prevent morbidity.^1^

**Figure 1:**
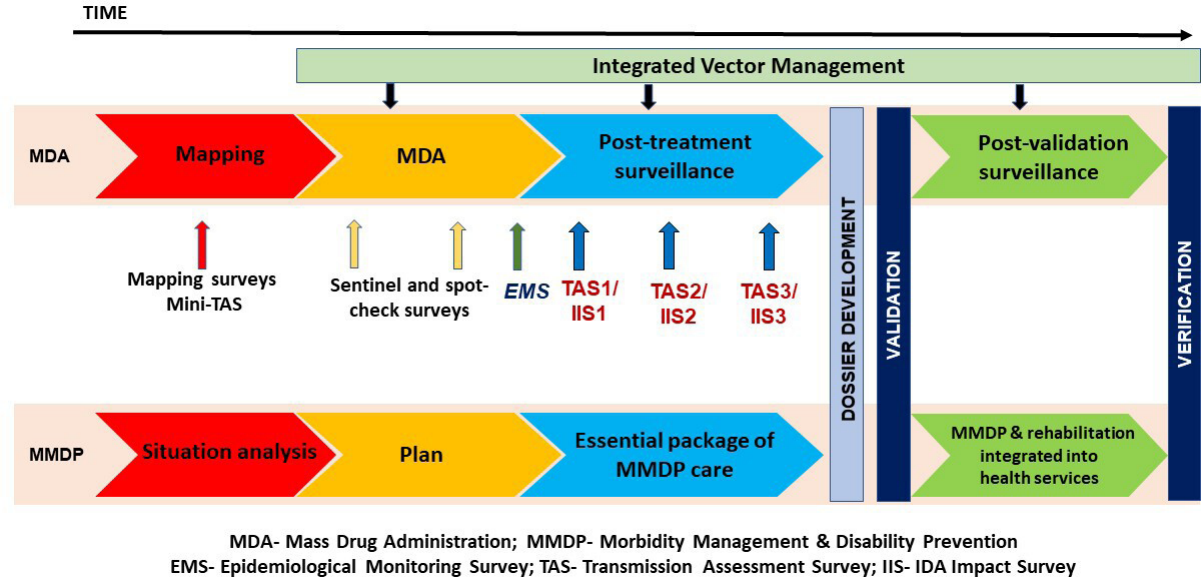
The GPELF strategic framework (based on information from WHO, 2025 & NCVBDC, 2024). GPELF, Global Programme to Eliminate Lymphatic Filariasis.

The GPELF strategic framework consists of a series of programme steps to implement, monitor and evaluate WHO recommended interventions towards achieving the LF elimination goals as depicted in figure 1. Accordingly, the programme aims for all endemic countries to complete MDA, conduct surveillance after stopping MDA, validate achieving EPHP and subsequently implement post-validation surveillance (PVS) integrated into existing healthcare systems.^1 3^

Briefly, to stop MDA (minimum five rounds of DA/IA or 2 rounds of IDA or 10 rounds with albendazole), each evaluation unit (EU) with a population less than 500 000 must ensure an effective coverage of 65% during each MDA round (irrespective of regimen used) and demonstrate microfilaria (Mf) prevalence below 1% or circulating filarial antigen (CFA) prevalence less than 2% at sentinel and spot-check sites after every round of MDA, which will be again confirmed through epidemiological monitoring surveys (EMS) previously called pre-transmission assessment survey (TAS).^1 4^ EUs that clear EMS will proceed to resource-intensive first TAS in areas under two drug regimen or IDA Impact Survey (IIS) in areas under triple drug regimen based on which MDA-stop decisions will be made. EUs that pass first TAS or IIS (Mf<1% or CFA<1%) will undergo second and third TAS/IIS at an interval of 2 years to confirm sustenance of transmission interruption and thus will be under post-MDA surveillance for 4–6 years. on passing of TAS-3/IIS-3, the EU enters the phase of PVS.^1^

Validation is defined as documentation of a country’s claim to have achieved EPHP through passing of three serial TAS/IIS as well as ensuring morbidity management and disability prevention is in place in all endemic areas/districts within the country and official recognition of their achievement by the WHO.^1^ Countries are encouraged to begin PVS after passing TAS-3 or IIS-3, even though validation is only conferred at the national level.^1^ The post-validation phase is a long-term activity involving surveillance strategies like health facility screening, integrated surveys through existing surveillance platforms for other diseases, molecular xenomonitoring (MX) and screening of high-risk areas and populations such as migrants, nomads, etc. The WHO advocates active surveillance combining any two of the above strategies to monitor periodically and confirm sustained interruption of transmission, detect residual foci if any, prevent resurgence and safeguard the disease-free status.^1^ Further, it recommends continuation of PVS for no less than 10 years after validation or if resources allow, with particular attention to regions that were previously under MDA as they are at high risk of recrudescence.^1^ Evidence from countries under PVS such as Sri Lanka and Tonga indicates that, even after achieving EPHP, local transmission can still continue and, if appropriate PVS strategies are not in place, can eventually result in resurgence.^5^,^8^ A cross-sectional study (2024) in the two kingdoms of Futuna, a Pacific island under PVS with a population of 3063 detected antigen prevalence of 2.5% in the kingdom of Alo and 7.5% in Taoa among children aged <18 years who were born after the last round of MDA in 2007.^9^ Although the long-term aim of PVS is to verify elimination of transmission, the parameters/ criteria to demonstrate zero transmission are yet to be defined.^1^

MDA has been implemented in 71 of 72 endemic countries since 2000. Through the collective efforts of the WHO and member nations, nearly 943.4 million people at risk of infection had received over 10 billion cumulative treatments which resulted in a 69.2% reduction in the population requiring MDA as of 2024.^2^ While 16 countries are currently under surveillance after stopping MDA, 35 are still continuing MDA and 21 countries achieved EPHP.^2^

In the South-East Asian region comprising nine endemic countries, five have achieved EPHP and more than 74% endemic areas in Nepal, Indonesia and Myanmar have stopped MDA. In India, which contributes to 63% of global burden (306 million at-risk population) with 345 endemic districts spread across 20 states and union territories, 161 districts are under MDA, 141 districts have cleared the first TAS and stopped MDA, with an additional 43 districts awaiting TAS.^2^ Most of the endemic districts under MDA are from the states of Bihar, Jharkhand and Uttar Pradesh.^10^ To accelerate progress, the country launched an enhanced five-pronged strategy in 2023, introducing new interventions for LF elimination.^11^

Currently in India, 106 endemic districts have cleared TAS3^11^ and therefore require continued surveillance to ensure transmission interruption as they are at risk of recrudescence due to the in-migration of individuals from endemic areas under MDA in search of livelihood. Evidence from a non-endemic area in Punjab state of India reported 2.01% Mf prevalence among migrants from endemic states, indicating that the migrants, if untreated, may remain a potential reservoir for transmission.^12^ However, the role of migrants in LF transmission has not yet been explored in India, highlighting the need to address and develop targeted intervention strategies to prevent transmission, if any. Further, no data are available on the current status of LF in the districts that have passed all three serial TASs. Particularly, when there is a lot of cross-border travel and mobility from the endemic districts currently under MDA, leading to resurgence or creation of new foci of transmission in these areas cannot be ruled out.

Hence, this study is proposed to assess the filarial infection prevalence (in humans and vectors) in three districts that have passed all three serial TAS. All these districts have high in-migration from endemic areas currently under MDA in the country. The study aims to measure filarial infection in (1) adult population aged 20 years and above among general population, (2) migrant population (aged 2 years and above) from endemic areas and (3) vectors using MX to confirm sustained transmission interruption or identify any potential risk of resurgence. In addition, in one MDA-naive district that shares borders with endemic districts, filarial infection status in (1) school children aged 9–14 years, (2) migrants (aged 2 years and above) from endemic areas and (3) vectors using MX will be assessed.

This study will be implemented by the Indian Council of Medical Research-Vector Control Research Centre (ICMR-VCRC), a WHO collaborating centre for research on LF in collaboration with the National Center for Vector Borne Diseases Control (NCVBDC) and state health programmes, with financial support from the Bill & Melinda Gates Foundation.

## Methodology

### Study settings

**Figure 2:**
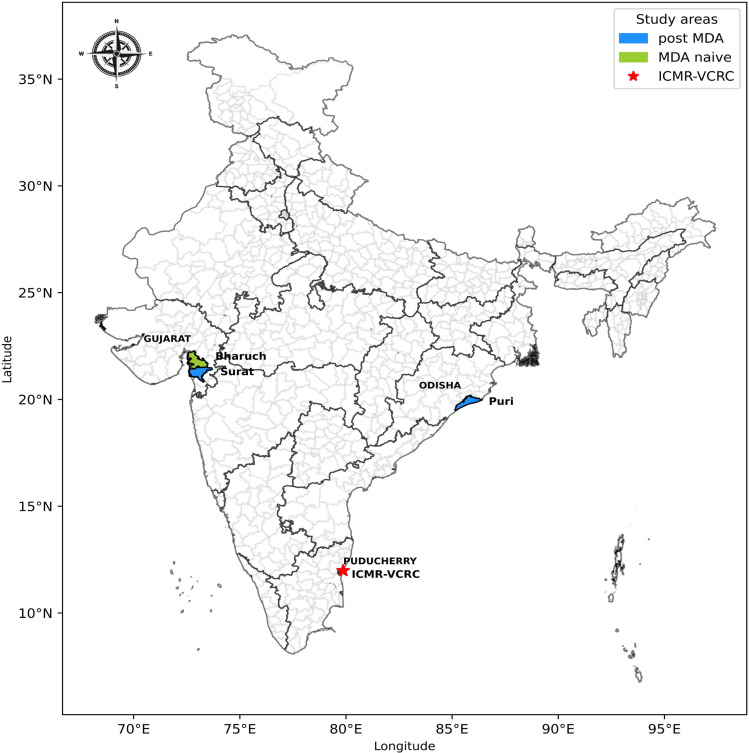
Map of India showing the selected study states and districts. ICMR-VCRC, Indian Council of Medical Research-Vector Control Research Centre; MDA, mass drug administration.

This cross-sectional study will be conducted in one EU from each of the three districts that have passed all three serial TASs and are in post-MDA phase: Puducherry, Surat (Gujarat) and Puri (Odisha). In addition, one EU will be selected in one MDA-naive district adjacent to Surat: Bharuch (Gujarat) (figure 2).

As per the national guidelines,^11^ an EU is an area with a population of approximately 500 000, which could be one or more administrative blocks in the selected district. The population details at the block and village or ward level, along with migrant holdings, will be collected from the health and labour departments of respective states. If necessary, geographically contiguous blocks will be combined to arrive at a population of 500 000 and considered as a single EU under each district. In one selected EU under the MDA-naïve district (1) a list of all schools with enrolment details of children aged 9–14 will be obtained from the concerned education department for the school surveys and (2) a list of high-risk sites based on the number of filariasis cases will be obtained.

The details of the four study areas are described in table 1.

**Table 1:** Details of the four study areas

District and state/Union Territory (UT)	Blocks/evaluation unit (EU)	Total population	Year of last MDA	Year of passing TAS1 and TAS3	Migrant population (2025)*
Puducherry district, Puducherry UT	Villiannur and Oulgaret	422 570	2011	2013; 2019	1148†
Puri, Odisha	Puri and Brahmagiri	440 618	2014	2016; 2021	Not available
Surat, Gujarat	Kamrej and Palsana	409 089	2016	2017; 2022	95 506
Bharuch, Gujarat	Bharuch	486 706	MDA-naive		60 443

* Source of the information: Health and Labour Departments of the respective districts.

† only organised sector.

MDA, mass drug administration; TAS, transmission assessment surveys.

### Selection of study clusters within EUs

In the three endemic EUs under post-MDA phase, 30 clusters (villages or wards) will be selected based on the probability proportional to estimated size (PPES) method, for conducting (1) community surveys (adults in general population and migrants) and (2) vector surveys for MX.

In the MDA-naïve EU (1) a school-based Mini-TAS (Mini-TAS) protocol will be implemented in 30 schools selected using the confirmatory mapping tool^1^; (2) a migrant survey will be conducted in 30 randomly selected clusters selected based on the PPES method and (3) vector surveys for MX will be carried out in five purposively selected high-risk clusters reporting a large number of filariasis cases.^13 14^

**Figure 3:**
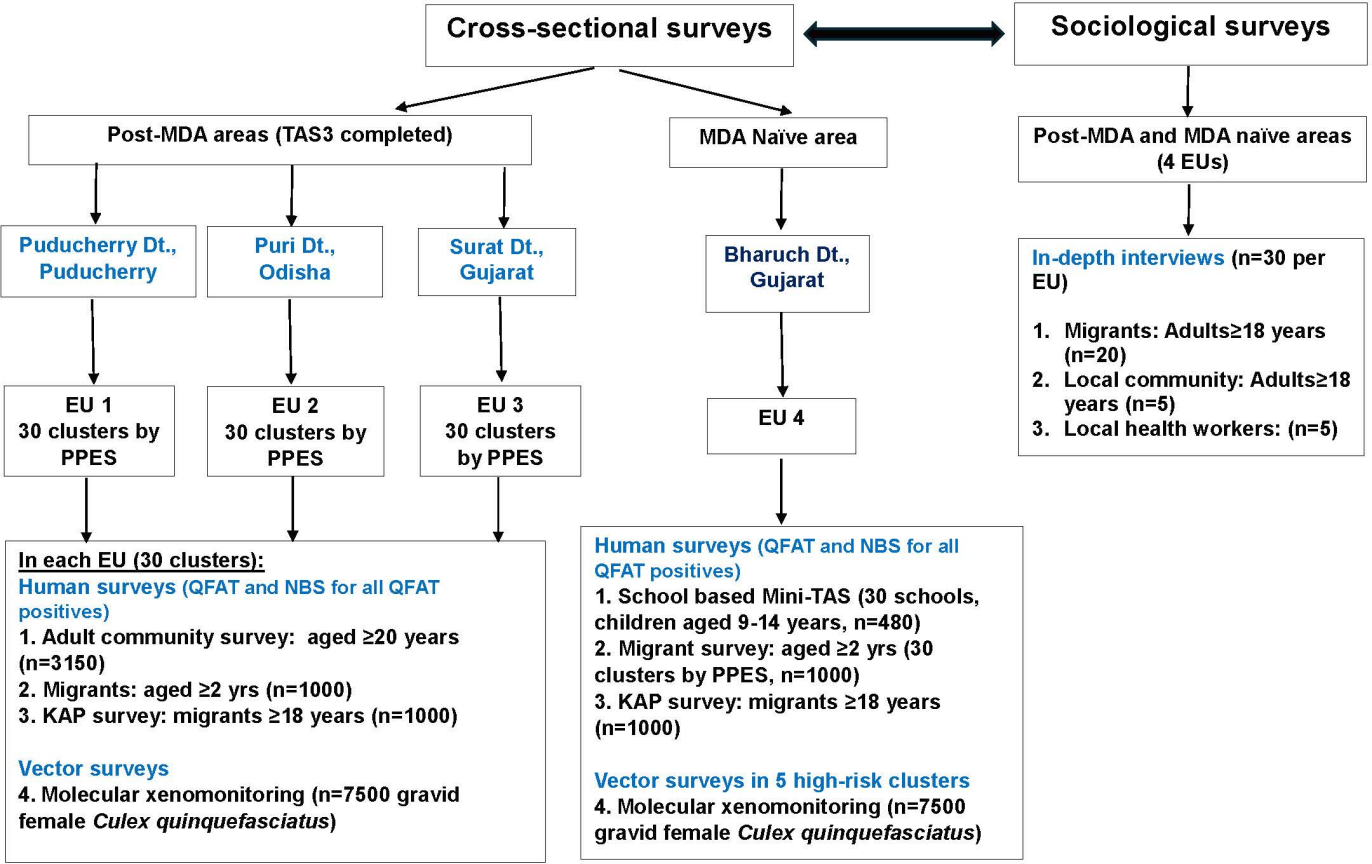
Flow chart showing the study methods. EUs, evaluation units; KAP, knowledge, attitude and practice; MDA, mass drug administration; NBS, Night blood smear; PPES, probability proportional to estimated size; QFAT, Q-filariasis antigen test; TAS, transmission assessment surveys.

The flow chart showing the study methods is depicted in figure 3.

### Study population, sample size and sampling methods

#### Adult community surveys

This will be conducted using the IIS protocol by WHO and NCVBDC in the three endemic EUs.^1 11^ Accordingly, 3150 adults aged ≥20 years from 30 clusters (105 per cluster) within each EU will be tested for CFA using Q-filariasis antigen test (QFAT)^1 15^ and those positive for QFAT, night blood smears will be collected for Mf as described in the bench-aid by WHO.^1^ Any individual residing in the EU for more than 1 year will be eligible to participate in the study and those terminally ill will be excluded.

The total number of households (HH) with approximate population in the selected cluster will be obtained from the respective Primary Health Centres (the first level of healthcare contact with the community). From the total population, the number of adults aged ≥20 years will be estimated. Accordingly, the number of HH to be sampled to achieve the required sample size of 105 per cluster will be estimated and the sampling interval will be calculated. Once done, the HHs will be selected systematically and all available adults at the time of the survey will be explained about the purpose of the study. Prior to testing, the survey team will obtain written informed consent to participate in the study and then they will be enrolled using a format on the smartphone as well as a handwritten proforma.

#### Migrant surveys

These surveys will be carried out in all the 4 EUs. The International Organization for Migration (part of the United Nations) defines a migrant as ‘a person who moves away from his or her place of usual residence, whether within a country or across an international border, temporarily or permanently, and for a variety of reasons’.^16^ As a part of this study, consultations were held with various stakeholders from the NCVBDC, LF experts, non-governmental organisations and implementers in the field to define migrants in the specific context of LF and MDA in India. The operational definition for a migrant keeping the biology and epidemiology of LF in context was finalised and is given below:

A person who has moved away from his or her place of usual residence in an LF endemic district currently under MDA and is now residing in the EU for a period ranging from 1 month to 10 years for various reasons, and who meets the following inclusion criteria:

1. Stayed in the EU for at least 1 month, considering the minimum incubation period of 4 weeks for LF as per the literature.^11^
2. Stayed in the EU for not more than 10 years, considering the fecundity and lifespan of the adult filarial worm.^17 18^

Any migrant in the study area who meets either of the above two criteria must have stayed for at least 1 month during his/her visits to their home-town (endemic district/state) to ensure adequate exposure and risk of infection.

Temporary and long-term migrants (either individual or family) will be excluded from the migrant survey. A temporary migrant is defined as a person who has resided in the study area for less than 1 month, while a long-term migrant is one who has resided in the study area for more than 10 years.

A minimum sample size of 1000 (per EU) was calculated for migrant surveys based on an expected Mf prevalence of 2.8% (pilot study by VCRC, unpublished) with a precision of 1.5%, design effect of 1.5 and an anticipated non-response rate of 30%. The survey will be conducted in the same 30 randomly selected clusters within the EUs, and the sample will be proportionately distributed according to the migrant population in each cluster.

Mapping of migrants’ holdings/settlements will be carried out with the help of the health and labour departments in each district. Within the selected cluster, eligible individuals will be identified, and the survey team will explain the purpose of the study and obtain informed written consent before enrolment. If necessary, prior permission will be obtained from the labour department and the employers to test the migrant. If the survey team comes across any migrant family, all household members aged above 2 years will be tested for filarial infection as described earlier. Information on the state of origin of the migrant and its current MDA status will be recorded using standard proformas. In addition, a knowledge, attitude and practice (KAP) questionnaire will be administered to gather information on their knowledge of LF, MDA and if not participated in the MDA in their native place, the reasons for the same.

#### School survey

In the MDA-naive EU, the Mini-TAS protocol^1^ will be implemented in which 480 school children aged 9–14 years (school grades 4–8) will be surveyed from 30 selected schools. Only those children residing in the EU will be eligible to participate. They will be tested for filarial infection (as described under community surveys) only after obtaining a written informed consent from the parent/legal representative and school principal.

#### Qualitative surveys

In-depth interviews (IDIs) will be conducted among migrants, community members and local health personnel at the selected EU. Specific interview guides will be developed for each group and used during the IDIs (figure 3). These interviews are expected to provide an in-depth understanding of the probable reasons for their infection status (non-participation in MDA, missing MDAs due to migration, etc), perception of LF risk and to devise targeted interventions for the migrants.

From each of the four study EUs, 20 migrants who test QFAT positive will be purposively selected for IDIs. Priority will be given to the cluster with the highest number of positive cases to maximise the relevance of the sample. The selection process will also consider diversity in age, gender and duration of migration to capture a range of experiences. Additionally, five IDIs will be conducted with local health workers (Accredited Social Health Activists, Auxiliary Nurse Midwives and Community Drug Administrators) at each site to gather their insights on migration patterns and LF transmission risks. To complement this, five IDIs will be carried out among community members at each study location to understand local perceptions of LF and migration.

#### MX surveys

The two-stage cluster sampling protocol (150 HHs×2 pools×25 gravid *Culex quinquefasciatus*=7500 mosquitoes from 30 clusters) developed and validated by ICMR-VCRC will be used in these surveys,^19 20^ assuming a provisional threshold of 0.25%. The objective is to assess vector infection levels, with a target of collecting 7500 gravid Culex quinquefasciatus mosquitoes per EU.

The mosquito survey will be carried out in two stages. In stage 1, mosquito collection will be conducted in the same 30 clusters selected for human blood surveys in the three endemic EUs as well as in five purposively selected high-risk clusters in the MDA-naïve EU. In stage 2, a total of 150 HHs will be systematically selected from all these clusters for mosquito trapping. In each selected HH, 2 pools of gravid *Culex quinquefasciatus* mosquitoes with a pool size of 25 each will be captured using gravid traps. The number of HHs to be sampled from each selected cluster will be proportional to the total number of HHs in those clusters. The details of the number of mosquitoes collected and the number of days spent on collection in each selected HH will be recorded. The mosquito pools will be processed using quantitative PCR assays for the detection of *Wuchereria bancrofti* DNA.

#### Procedure for trapping of mosquitoes

Verbal permission will be obtained from the head of the selected household prior to placement of traps. Gravid traps containing an infusion to attract gravid *Culex quinquefasciatus* mosquitoes will be placed in selected HH. Traps will be placed in suitable outdoor locations before 18:00 hours and will be removed the next day before 08:00 hours (this will be repeated for a maximum of 3 days) and taken to the lab for further processing. A temporary field laboratory will be set up in each EU, where a trained entomologist will identify and segregate the mosquito species. Gravid *Culex quinquefasciatus* mosquitoes (fully fed, semigravid or gravid) will be transferred into two separate, uniquely barcoded vials labelled A and B. These vials will be placed in a dry bath for 24 hours (to prevent fungal contamination). The procedure will be repeated for the 3 days of collection or till both the vials have 25 *Culex quinquefasciatus* gravids, whichever is earlier. A proforma will be used to collect the details of the areas surveyed, household identification number, number of days spent for mosquito collection in each household, geo-coordinates of the selected household, the number of mosquitoes collected in each pool, and their gravid status. Once processed, the sealed vials will be transported bi-monthly to headquarters for molecular processing.

#### PCR assays to determine vector infection

Filarial parasite DNA will be extracted from each mosquito pool using the commercial DNeasy blood and tissue kit (Qiagen, Hilden, Germany). This includes grinding the mosquitoes using steel beads in a Tissue Lyser II (Qiagen) for 15 min at 25 frequency/sec and adding proteinase-K. The extracted DNA samples will be coded and analysed by real-time quantitative PCR as described elsewhere.^19 21^ Each PCR reaction will be performed with 12.5 µL of FastStart Essential DNA probes Master (Roche Diagnostics, Germany), 450 nmol/L of each primers: LDR1-5’ATTTT GATCATCTGGGAACGTTAATA-3’; LDR2-5’CGACTGTCTAATCCATTCAGAGTGA-3’ and 125 nmol/L probe (6 FAM-ATCTGCCCATAGAAATAACTACGGTGGATCTG-TAMRA) (IDT, USA) in a final volume of 25 µL in 96-well MicroAmp optical plates (Roche Diagnostics, Germany). A 1 μL of the extracted DNA will be used as the template in each real-time PCR as described earlier, along with 1 ng, 100 pg and 10 pg of purified genomic DNA samples as positive controls and water controls. All reactions will be run in duplicates by trained technicians.

Thermal cycling will be performed at 50°C for 2 min, 95°C for 10 min, followed by 40 cycles of 95°C for 15 s and 60°C for 1 min. Thermal cycling and data analysis will be done with the CFX96 Real-Time System (Bio-Rad). Cq values of samples ranging from 1.0 to 39.0 will be considered positive, and samples that failed to reach the fluorescence threshold beyond 39.0 will be considered indeterminate and repeated to confirm the negativity or positivity of those samples as described by Rao *et al*.^22^ The processed results will be used to assess the pool positivity status, which subsequently will be used to estimate the Wb parasite DNA prevalence using the R program ‘PoolTestR’ for the EU.

### Training of project staff

The field teams will be adequately trained prior to the initiation of the surveys. The training for blood survey teams will cover informed consent procedures, enrolment documentation, finger-prick blood collection techniques, use of QFAT kits, smear preparation and staining, and handling of samples. Vector survey training will include infusion preparation, trap assembly and deployment, battery maintenance, mosquito identification, use of dry baths for sample processing, labelling and storage in vials, and procedures for transportation to the central laboratory for PCR assays. A trained sociologist will train the staff in conducting qualitative interviews.

For blood surveys, two field teams will be deployed in each study EU, with each team comprising 3–4 trained members responsible for specific tasks. One member will obtain informed consent from eligible participants and enrol them; another will prepare QFAT kits or slides and read test results; a third member will collect finger-prick blood samples; and a fourth member will assist in digital data entry via smartphones. Vector surveys will be conducted by a two-member entomology team in each EU. This team will prepare mosquito attractant infusions using hay, malt and yeast fermented over 5–6 days, assemble gravid traps and deploy traps in selected HHs. These staff will be hired locally to ensure familiarity with the local context and language. To ensure quality in survey procedures, a 2-day training programme will be conducted for all field staff.

### Data management and quality control

Electronic data collection will be carried out using smartphones equipped with the REDCap software for real-time data capture. The principal investigator and coinvestigators will closely monitor data collection daily and check for any errors in the data throughout the study. Each QFAT test and corresponding blood smear slide will be tagged with a unique barcode label to ensure accurate tracking and linkage of individual-level data. For vector surveys, unique barcode IDs will be assigned to each household and to the two mosquito pools (A and B) collected per household, and these identifiers will also be entered in the mobile REDCap app to maintain data integrity. QFAT test results will be read independently by both the designated card reader and the team leader. In the case of any discrepancy between their readings, a third opinion will be sought to finalise the result. Blood smear slides will be examined by two independent technicians, and all the positive slides and 10% of negative slides will be cross-examined by a senior technical staff member to ensure quality.

### Data analysis plan

R software will be used for analysis of the data. PoolTestR will be used to analyse the vector data. EU-wise Mf/CFA prevalence and vector infection rates with 95% CIs will be estimated. Even if the overall estimates are well below the elimination thresholds, if the lower CI exceeds the threshold limit, this would indicate the probable risk which would require surveillance. The risk associated with LF positivity (CFA/Mf) will be assessed using multivariate logistic regression analysis among the general and the migrant population. Recorded IDIs will be transcribed verbatim to English and thematically analysed using Atlas-ti software.

### Study status

The study is approved for a period of 2 years (November 2024- November 2026). Administrative approvals and permissions were obtained by July 2025, following which the study was initiated in one of the sites in August 2025.

### Key output indicators

The key output indicators of the study are shown in table 2.

**Table 2:** Key output indicators

Phase of LF elimination	Study method	Output indicators	Interpretation (at EU level)
Post-MDA	Community blood surveys in 30 clusters among adults aged ≥20 years (n=3150 per EU)	Mf prevalence CFA prevalence	Mf ≥1% and CFA ≥1% indicates ongoing transmission
Post-MDA	Vector surveys in 30 clusters (n=7500 per EU)	Wb Parasite DNA prevalence	If ≥0.25% indicates ongoing transmission
MDA-naive	School survey (n=480)	CFA prevalence	If >3 CFA positives, EU will be considered endemic.
MDA-naive	Vector surveys in 5 high-risk clusters (n=7500)	Wb Parasite DNA prevalence	If ≥0.25% indicates ongoing transmission, EU is endemic
Post-MDA and MDA-naive	Migrant blood survey with KAP and Qualitative in-depth interviews	Mf prevalence CFA prevalence Sociodemographics and awareness on LF	Magnitude of infection will be known and factors associated with transmission in context of migrants will be known

CFA, circulating filarial antigen; EU, evaluation unit; KAP, knowledge, attitude and practice; LF, lymphatic filariasis; MDA, mass drug administration; Mf, microfilaria.

## Discussion

Currently in India, 106 districts have passed all three serial TASs and many of these districts had stopped MDA several years back after passing TAS1.^10^ A few states like Tamil Nadu are in the process of preparing and submitting dossiers for validation of achieving EPHP.^23^ While Puducherry district, an erstwhile French colony and a tourist destination^24^ and also thriving as an industrial hub, shares a border with the state of Tamil Nadu, it had stopped MDA 12 years ago,^10^ Surat (a textile and diamond hub)^25^ and Puri (a tourist destination in east India)^26^ districts had stopped MDA 8 and 9 years ago,^10^ respectively, after passing of TAS. However, all three selected districts have high in-migration from the high LF endemic districts in the states of Bihar, Jharkhand and Uttar Pradesh in search of better livelihood.^10 27^ Although these districts are currently under the post-MDA surveillance, the LF situation in these areas is not known since the stopping of MDA.

Human mobility has long been an underexplored determinant of LF transmission dynamics.^28 29^ In areas where competent vectors exist, infected migrants can act as reservoirs for re-establishing transmission.^29^ In cross-border areas where the vector-parasite combination is favourable to transmission, they may undermine the outcomes of elimination programmes.^14 28 30^ Studies have shown that migrants, if untreated, may remain a potential reservoir for transmission, especially in those districts that are in post-MDA phase or those that are MDA naïve.^31 32^ Countries like Togo, Malaysia and Sri Lanka have reported that migrants are a cause of concern as the countries approach the targets of LF elimination.^30 31 33^

The present study proposes to see the current status of LF transmission in the selected EUs that have stopped MDA and assess the risk of probable transmission, if any, using the human and vector infection indicators. The integration of molecular xenomonitoring (MX) alongside human surveys represents a major strength of the study. MX has emerged as a sensitive, non-invasive tool capable of detecting low-level transmission that may be missed by TAS, particularly in low-prevalence or post-MDA contexts.^2033^,^35^ TAS, a decision-making tool recommended by the WHO, has shown limited sensitivity in identifying focal recrudescence, particularly in very low prevalence areas (Mf<1%).^1^ In such situations, by directly testing vectors, MX surpasses the lag inherent in detection of human antigen or Mf.^36^ In view of the high sensitivity of MX, a study in Burkina Faso has shown that an integrated MX with existing surveillance platforms like that of malaria will not only be highly useful and cost-efficient in post-MDA situations but also a promising strategy for the future.^37^

In addition, the present study will also assess the LF situation in an adjoining MDA-naive area which will help to document any risk of transmission considering its proximity to endemic areas. Risk of transmission in areas bordering endemic districts was reported by a study in Salem district of Tamil Nadu, an MDA-naive area.^14^ The authors suggested carrying out blood surveys and/or MX particularly in bordering areas.^14^ Mini-TAS has been recommended for mapping unsurveyed/MDA-naive areas by WHO and literature too supports this recommendation.^1 14 38^ Yet another strength of this study can be attributed to the mixed method design which combines surveys for detecting filarial infection in humans and vectors along with qualitative surveys among migrants to get a holistic view of the epidemiological situation and in-depth understanding of the challenges with respect to migrants.

As the selected EUs have high in-migration from the LF endemic states, this will be the first study to document the infection levels among these migrants, identify the factors associated with positivity and the reasons for non-participation/missing the MDA rounds through cross-sectional blood surveys and qualitative interviews. These findings will aid in better understanding of the LF situation and are expected to guide the programme to devise targeted strategies to bring these migrants within the ambit of MDA so that they no longer stay as potential reservoirs for infection.

### Limitations

Certain challenges are anticipated in the study. As MDA has been stopped for many years, people may not be able to accurately recollect details about MDA or their participation in the rounds, resulting in potential recall bias. It is anticipated that the community may not come forward to provide blood samples for testing for filariasis (as MDA is stopped, there may be a low risk perception for the disease in the community). To overcome this, prior information on the survey will be provided through the local primary health centres which cater to the villages and local workers like accredited social health activists and other health workers (ground level workers known/familiar to the community) will be involved during the filarial blood surveys for community sensitisation and mobilisation. Engaging migrant populations, who are often mobile and hard to reach, may pose logistical and operational difficulties. Identifying the migrant holdings and ensuring their participation in the study will require close coordination with local authorities, community health workers and employers. Additionally, attributing the risk of transmission to migrants may be complex and will necessitate careful interpretation of entomological and epidemiological data.

## Ethics and dissemination

Approval has been obtained from the ICMR-VCRC Scientific Advisory Committee and Institutional Human Ethics Committee (IHEC 03-0824/N/F). The proposal was critically reviewed by independent experts from the funding agency prior to final approval.

Written informed consent will be obtained from all willing participants prior to enrolment in the study. Parental consent will be obtained for all children aged <18 years and verbal/written assent will be obtained from children aged 7–17 years. All filarial infection positive individuals will be treated as per the national guidelines by the district health authorities. Study findings will be shared with all stakeholders through dissemination workshops and will be published in reputed open-access journals.
