# Supplementary material for: Cross-sectional epidemiological assessment of lymphatic filariasis situation in areas under post-mass drug administration surveillance and the associated risk of transmission in the context of migrants in India: a study protocol
Source: BMJ Open. 2026 Jan 30;16(1):e116111. doi: 10.1136/bmjopen-2025-116111 (PMC12863371; doi:10.1136/bmjopen-2025-116111)
Supplement: Supplementary file 1 [file bmjopen-2025-116111.reviewer_comments.pdf]

PEER REVIEW HISTORY

BMJ Open publishes all reviews undertaken for accepted manuscripts. Reviewers are asked to complete a checklist review form and are provided with free text boxes to elaborate on their assessment. These free text comments are reproduced below.

ARTICLE DETAILS

Title (Provisional)

A cross-sectional epidemiological assessment of Lymphatic filariasis situation in areas in post-Mass Drug Administration phase and factors associated with risk of transmission in context of migrants in India-a study protocol

Authors

Srividya, Adinarayanan; Dinesh, Raja J; M M, Muhammed Jabir; Kishanthini, Manikandan; Dogra, Vishal; Tripathi, Bhupendra; Sharma, Rinku; Jain, Tanu; Rahi, Manju

---

This article was not externally reviewed at BMJ Open. Protocols that have been independently assessed prior to submission to BMJ Open are usually fast-tracked to publication on the grounds that further substantial changes will not be possible. This independent assessment will usually be external, independent review for both a substantial grant award from a non-commercial or government funder and ethics approval.
